# Identification of *Leptospira* serovars by RFLP of the RNA polymerase beta subunit gene (*rpo*B)

**DOI:** 10.1590/S1517-838246220120018

**Published:** 2015-06-01

**Authors:** Lenice Roteia Cardoso Jung, Maria Rosa Quaresma Bomfim, Erna Geessien Kroon, Álvaro Cantini Nunes

**Affiliations:** 1 Universidade Federal de Minas Gerais, Departamento de Biologia Geral, Instituto de Ciências Biológicas, Universidade Federal de Minas Gerais, Belo Horizonte, MG, Brasil, Departamento de Biologia Geral, Instituto de Ciências Biológicas, Universidade Federal de Minas Gerais, Belo Horizonte, MG, Brazil.; 2 Universidade Federal de Minas Gerais, Departamento de Microbiologia, Instituto de Ciências Biológicas, Universidade Federal de Minas Gerais, Belo Horizonte, MG, Brasil, Departamento de Microbiologia, Instituto de Ciências Biológicas, Universidade Federal de Minas Gerais, Belo Horizonte, MG, Brazil.; 3 Universidade do Ceuma, Departamento de Biologia Parasitária, Universidade Ceuma, São Luis, MA, Brasil, Universidade Ceuma, Departamento de Biologia Parasitária, São Luis, MA, Brazil.

**Keywords:** *Leptospira*, rpoB gene, RFLP, serovar, DNA typing

## Abstract

Leptospires are usually classified by methods based on DNA-DNA hybridization and the conventional cross-agglutination absorption test, which uses polyclonal antibodies against lipopolysaccharides. In this study, the amplification of the *rpo*B gene, which encodes the beta-subunit of RNA polymerase, was used as an alternative tool to identify *Leptospira.* DNA extracts from sixty-eight serovars were obtained, and the hypervariable region located between 1990 and 2500-bp in the *rpo*B gene was amplified by polymerase chain reaction (PCR). The 600-bp amplicons of the *rpo*B gene were digested with the restriction endonucleases *Taq*I, *Tru*1I, *Sau*3AI and *Msl*I, and the restriction fragments were separated by 6% polyacrylamide gel electrophoresis. Thirty-five fragment patters were obtained from the combined data of restriction fragment length polymorphism (PCR-RFLP) analysis and used to infer the phylogenetic relationships among the *Leptospira* species and serovars. The species assignments obtained were in full agreement with the established taxonomic classifications. Twenty-two serovars were effectively identified based on differences in their molecular profiles. However, the other 46 serovars remained clustered in groups that included more than one serovar of different species. This study demonstrates the value of RFLP analysis of PCR-amplified *rpo*B as an initial method for identifying *Leptospira* species and serovars.

## Introduction

Leptospirosis is a zoonotic disease of global importance that has emerged as a major cause of morbidity and mortality among impoverished populations (Ko *et al.*, 2009). Based on global data, more than 500,000 new cases of leptospirosis are reported annually, with mortality rates exceeding 10% (WHO, 1999, 2006). Multiple factors, including environmental, demographic, social, and economic factors, have contributed to the emergence of this disease, which affects a broad range of mammalian hosts, including humans, wildlife, and domestic animals (Bharti and Nally, 2003; Lau *et al.*, 2012).

The precise identification and classification of *Leptospira* spp. is important for epidemiological and public health surveillance (Mohammed *et al.*, 2011). Leptospires are usually classified by methods based on DNA-DNA hybridization, whereas the cross-agglutination absorption test (CAAT), which uses polyclonal antibodies against lipopolysaccharides (LPSs), has led to the definition of serovars that are today considered to be the basic systematic units of *Leptospira* spp. (Cerqueira and Picardeau, 2009; Galloway and Levett, 2010). Serological methods for the characterization of *Leptospira* species are complex and costly, restricting their worldwide distribution and use (Ahmed *et al.*, 2010).

Many molecular DNA techniques have been applied to identify and classify the species and serovars of *Leptospira* (Ahmed *et al.*, 2012). These include restriction endonuclease analysis (REA) of chromosomal DNA (Marshall *et al.*, 1981), random amplified polymorphic DNA (RAPD) fingerprinting (Ramadass *et al.*, 1997), DNA-DNA hybridization (Yasuda *et al.*, 1987; Brenner *et al.*, 1999), arbitrarily primed PCR (Ramadass *et al.*, 2002), pulsed-field gel electrophoresis (PFGE) (Galloway and Levett, 2008) and polymerase chain reaction (PCR) of specific genes followed by restriction fragment length polymorphism analysis (RFLP) (Li *et al.*, 2009). Recently, multilocus sequence typing has been applied as an alternative to immunological methods for the identification and classification of pathogenic leptospires (Ahmed *et al.*, 2006; Pavan *et al.*, 2008; Leon *et al.*, 2010; Ahmed *et al.*, 2011; Boonsilp *et al.*, 2013). All of these techniques mentioned above have contributed significantly to the current taxonomic classification of the *Leptospira* genus (Morey *et al.*, 2006; Slack *et al.*, 2009).

Quantitative DNA-DNA hybridization to measure genetic homology has been used as a reference for the classification of serovars within species (Yasuda *et al.*, 1987; Perolat *et al.*, 1998, Brenner *et al.*, 1999). However, this hybridization method is not routinely used for the identification of *Leptospira* species due its complex and laborious execution, which requires the use of radioactive isotopes and is therefore restricted to reference laboratories (Morey *et al.*, 2006). It has also been observed that some serotypes are more characteristic of a single species, while others contain both pathogenic and nonpathogenic serovars (Morey *et al.*, 2006). Furthermore, little correlation has been shown between serological classification and genotypic systems because a given serogroup can often be found in several species of *Leptospira* (Ahmed *et al.*, 2012).

In addition to DNA-DNA hybridization and the other molecular methods mentioned above, specific PCR amplification of the 16S rRNA gene has contributed to the molecular characterization of some species of *Leptospira* (Ahmed *et al.*, 2012). The advantage of this technique is that the use of a DNA template, particularly one designed based on the region that encodes the bacterially conserved 16S rRNA gene, can clearly reveal phylogenetic relationships among species (Morey *et al.*, 2006).

La Scola *et al.* (2006a) have designed a universal primer pair for the identification of *Leptospira* species based on the gene encoding the β subunit of RNA polymerase (*rpo*B). These primers have been used to amplify and sequence the partial *rpo*B gene from 16 *Leptospira* species. According to the authors, analysis of the *rpo*B gene "may be useful as an initial screening test for the serovar identification of a new isolate of *Leptospira* and the detection or identification of *Leptospira* in clinical or environmental samples".

In previous studies, the utility of the *rpo*B gene for spirochete distinction among various bacterial species has been demonstrated (Renesto *et al.*, 2000; Lee *et al.*, 2000; Khamis *et al.*, 2004; Balamurugan *et al.*, 2013). Thus, the aim of this study was to investigate whether the PCR-amplified fragment of *rpo*B in conjunction with RFLP would allow for the determination of *Leptospira* serovars.

## Material and Methods

### Bacterial strains, media and growth conditions

For this study, sixty-eight *Leptospira* strains (Table 1) belonging to 11 reference species from the Pan American Institute for Food Protection and Zoonosis (INNPAZ) were used. Leptospires were grown for approximately five days at 30 °C in Ellinghausen-McCullough-Johnson-Harris (EMJH) culture medium (Difco) (Ellinghausen, 1973).

**Table 1:** The strains, serogroups, serovars, and species of the *Leptospira* genus used in this work.

Species	Serogroup	Serovar	Strain	Number
*L. biflexa*	Andamana	Andamana	CH11	1
	Semaranga	Patoc	Patoc I	2
*L. borgpetersenii*	Autumnalis	Srebarna	1409/69	3
	Ballum	Ballum	Mus 127	4
	Bataviae	Moldaviae	114-2	5
	Celledoni	Withcombi	Withcomb	6
	Hebdomadis	Nona	Nona	7
	Hebdomadis	Worsfoldi	Worsfoldi	8
	Icterohaemorrhagiae	Tonkini	LT 96-68	9
	Javanica	Javanica	Veldrat bataviae 46	10
	Mini	Mini	Sari	11
	Pyrogenes	Kwale	Julu	12
	Sejroe	Sejroe	M 84	13
	Tarassovi	Tarassovi	Perepelicin	14
*L. inadai*	Canicola	Malaya	H6	15
	Panama	Mangus	TRVL 137774	16
	Tarassovi	Kaup	LT 64-68	17
*L. interrogans*	Australis	Australis	Ballico	18
	Australis	Muenchen	Munchen C90	19
	Autumnalis	Autumnalis	Akiyami A	20
	Djasiman	Djasiman	Djasiman	21
	Bataviae	Bataviae	Van Tienen	22
	Canicola	Canicola	Hond Utrech IV	23
	Djasiman	Sentot	Sentot	24
	Gryppotyphosa	Valbuzzi	Valbuzzi	25
	Hebdomadis	Hebdomadis	Hebdomadis	26
	Icterohaemorrhagiae	Icterohaemorrhagiae	RGA	27
	Louisiania	Lanka	LT 25-67	28
	Mini	Szwajizak	Szwajizaki	29
	Pomona	Pomona	Pomona	30
	Pyrogenes	Pyrogenes	Salinem	31
	Sarmin	Waskurin	LT 63-68	32
	Sejroe	Hardjo	Hardjoprajitno	33
*L. kirschneri*	Australis	Ramisi	Musa	34
	Bataviae	Djatzi	HS 26	35
	Canicola	Bafani	Bafani	36
	Cynopteri	Cynopteri	3522 C	37
	Gryppotyphosa	Gryppotyphosa	Moskva V	38
	Hebdomadis	Kambale	Kambale	39
	Icterohaemorrhagiae	Mwogolo	Mwogolo	40
	Pomona	Mozdok	5621	41
*L. meyeri*	Mini	Parameles	Bandicoot 343	42
	Ranarum	Ranarum	Ranaram ICF	43
	Semaranga	Semaranga	Veldrat Semaranga	44
*L. noguchii*	Autumnalis	Fortbragg	Fort Bragg	45
	Djasiman	Huallaga	M7	46
	Panama	Panama	CZ 214K	47
	Pyrogenes	Myocastoris	LSU 1551	48
	Shermani	Carimagua	9160	49
*L. santarosai*	Autumnalis	Alice	Alice	50
	Bataviae	Kobbe	CZ 320K	51
	Cynopteri	Tingomariensis	M13	52
	Gryppotyphosa	Canalzonae	CZ188K	53
	Hebdomadis	Maru	CZ 285B	54
	Javanica	Vargonicas	24	55
	Mini	Georgia	LT 117	56
	Pomona	Tropica	CZ 299U	57
	Pyrogenes	Alexi	HS 616	58
	Sarmin	Weaveri	CZ 390U	59
	Sejroe	Trinidad	TRVL 34056	60
	Shermani	Luis	M6	61
	Tarassovi	Bakeri	LT 79	62
*L. weilli*	Celledoni	Celledoni	Celledoni	63
	Javanica	Coxi	Cox	64
	Sarmin	Sarmin	Sarmin	65
	Tarassovi	Vughia	LT 89-68	66
*L. terpstrae*	Icterohaemorrhagiae	Hualien	LT 11-31	67
*L. yanagawae*	Semaranga	Sao paulo	Sao paulo	68

### Isolation of DNA

An one-mL aliquot of each *Leptospira* serovar was cultured in 5 mL EMJH medium for 7 to 10 days at 30 °C. The culture was then centrifuged at 3000 × *g* for 30 min, and DNA was extracted from the bacterial pellet by adding 1 mL lysozyme solution (10 mg/mL in TE buffer (10 mM Tris and 1 mM EDTA, pH 8.0) and Wizard Genomic DNA Purification System reagents according to the manufacturer's instructions (Promega Co.).

### PCR assays

PCR amplification of a 600-bp region of the *rpo*B gene was performed with the primers 1900F (5′-CCTCATGGGTTCCAACATGCA-3′) and 2500R (5′-CGCATCCTCRAAGTTGTATTWCC-3′) as described by La Scola *et al.* (2006a). Each PCR reaction contained 1.5 mM MgCl_2_, 200 μM dNTPs, 25–50 ng of DNA template, 1.5 units of *Taq* DNA polymerase, and 50 pmol of each primer. The PCR amplification reactions were carried out in a Veriti 96-well Thermal Cycler (Applied Biosystems) under the following conditions: an initial denaturation step of 2 min at 95 °C, 33 cycles of denaturation for 30 s at 94 °C, annealing at 51 °C for 30 s and extension at 72 °C for 2 min, with a final primer extension step for 10 min at 72 °C.

### Restriction fragment length polymorphism (RFLP) analysis

To select enzymes for RFLP analysis, the results from *in silico* restriction digestion of twenty five *rpo*B sequences in GenBank^®^ were analyzed with Webcutter 2.0 program (http://bio.lundberg.gu.se/cutter2/) to distinguish the generated fragments following separation by 6% polyacrylamide gel electrophoresis. The genomic sequences used were as follows: AE016823.1, *L. interrogans* serovar Copenhageni str. Fiocruz L1-130; AE010300.2, *L. interrogans* serovar Lai str. 56601; CP000350.1, *L. borgpetersenii* serovar Hardjo-bovis strain JB197; and CP000777.1, and *L. biflexa* serovar Patoc strain Patoc 1 (Ames). DNA sequences of the *rpo*B gene reported by La Scola *et al.* (2006a) and sequences obtained by us in this study were also used. These sequences were deposited in GenBank^®^ under the accession numbers EU747300.1, EU747301.1, EU747302.1, EU747303.1, EU747304.1, EU747305.1, EU747306.1, EU747307.1, EU747308.1, EU747309.1, EU747310.1, EU747311.1, EU747312.1, EU747313.1, EU747314.1, EU747317.1, and EU747299.1, corresponding to *L. interrogans* serovar Bratislava, *L. kirschneri* serovar Grippotyphosa, *L. borgpetersenii* serovar Ballum, *L. interrogans* serovar Hardjo-prajitno, *L. interrogans* serovar Hebdomadis, *L. borgpetersenii* serovar Hardjo-bovis, *L. interrogans* serovar Pomona, *L. borgpetersenii* serovar Tarassovi, *L. interrogans* serovar Wolffi, L. biflexa serovar Andamana, *L. borgpetersenii* serovar Castellonis, *L. borgpetersenii* serovar Sejroe, *L. interrogans* serovar Djasiman, *L. interrogans* serovar Schueffneri, *L. borgpetersenii* serovar Whitticombi, *L. interrogans* serovar Sentot, and *L. interrogans* serovar Canicola, respectively.

PCR products were subjected to restriction digestion with *Taq*I, *Tru*1I, *Sau*3AI and *Msl*I endonucleases (Promega Co.) for 3 h at the recommended temperatures. To calculate the relative molecular masses of the digested fragments, a 100-bp DNA Ladder was used (Promega Co.). The digestion and separation of the DNA fragments by 6% polyacrylamide gel electrophoresis were repeated at least three times for all serovars to establish the final restriction patterns.

### Dendrogram construction

LabImage version 2.7.0 software (Kapelan GMBH) was used for constructing a binary matrix scored on the presence (1) or absence (2) of each fragment generated by PCR-RFLP with the *rpo*B primers. Cluster analysis based on similarity (Nei, 1972) was performed by the unweighted pair group method (UPGMA) with the arithmetic averages clustering algorithm (Sneath and Sokal, 1973), and the randomization procedure implemented in Tools for Population Genetic Analysis (TFPGA) software package according to Miller (1998) was used to construct the dendrogram.

## Results

*In silico* analysis of *rpo*B sequences deposited in GenBank indicated that a combination of four possible restriction enzymes was necessary to distinguish the *Leptospira* serovars as follows: *Taq*I, *Tru*1I, *Sau*3AI and MslI. Alone, each enzyme was able to identify only one or two different serovars.

**Figure 1:**
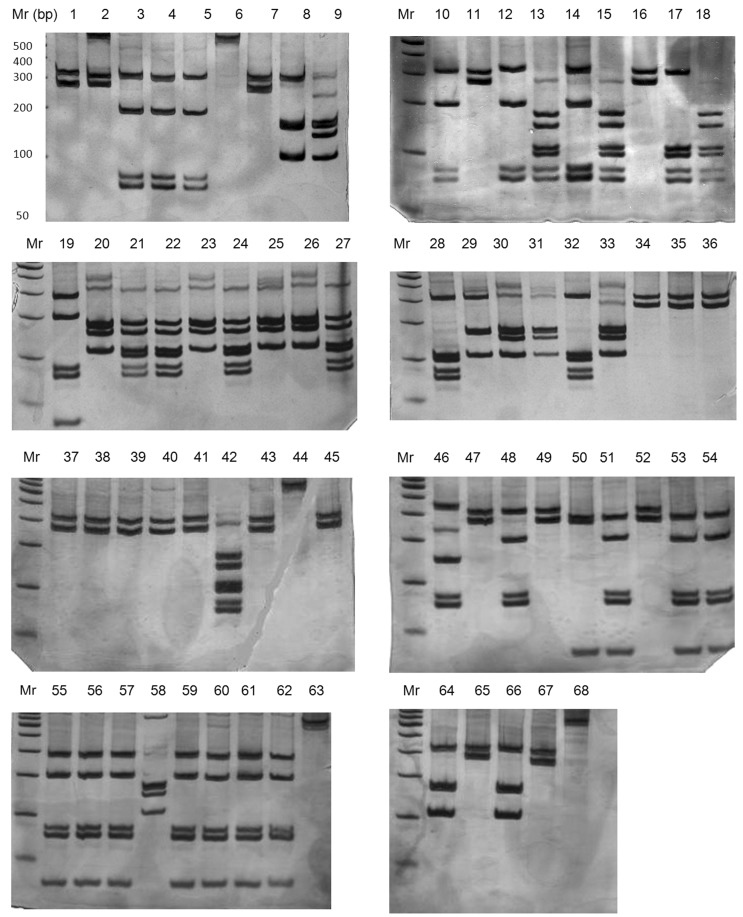
Polyacrylamide gel electrophoresis (6%) of the PCR products resulting from the digestion of the *rpo*B gene with the restriction endonuclease *Taq*I. was consistent with the 100-bp molecular weight ladder.

Digestion with *Taq*I resulted in ten different patterns (A to J), which are schematically represented in Table 2 and had the following frequencies: A, 29.4% (20); B, 10.3% (7); C, 7.35% (5); D, 13.2% (9); E, 11.8% (8); F, 4.41% (3); G, 16.2% (11); H, 7.35% (3); I, 1.47% (1); and J, 1.47% (1). Thus, *Taq*I identified two serovars, Huallaga of *L. noguchii* (profile I) and Alice of *L. santarosai* (profile J), as shown in Figure 1. The G profile pattern was observed in almost all *L. santarosai* serovars, with the exception of the serovar Alexi (profile D), and it was only identified in the Muenchen serovar *L. interrogans*.

**Table 2:** Restriction patterns of the 600-bp fragment of the *rpo*B gene of *Leptospira* following digestion with *Taq*I, *Tru*1I, *Sau*3AI, and *Msl*I endonucleases.

Pattern	Fragment size (bp)						
*Taq*I							
A	315			277			
B	315			198			
C	315			104	173	10	
D	144	171		104	173		
E	144	171		104	94	69	10
F	315			104	94	69	10
G	277		38	198		69	10
H	592						
I	144	369				69	10
J	277		38	277			
*Tru*1I Profiles							
A	41	166	33	352			
B	240			240			112
C	480						112
D	207		33	352			
E	240			352			
F	240			39	313		
G	592						
H	279				313		
I	41	199		352			
J	207		33	240			112
*Sau*3AI							
A	153	312					129
B	252		24	108	208		
C	153	99	24	108	208		
D	416					176	
E	276			108	32	176	
F	384				32	176	
G	276			140		176	
H	153	123		108	32	176	
I	252		132		79		129
*Msl*I							
A	592						
B	161		431				
C	126		466				
D	140		452				
E	317				278		

The *Tru*1I enzyme also exhibited ten distinct restriction patterns (A to J) with the following frequencies: A, 2.94% (2); B, 23.5% (16); C, 16.2% (11); D, 11.8% (8); E, 22.1% (15); F, 14.7% (10); G, 2.94% (2); H, 2.94% (2); I, 1.47% (1); and J, 1.47% (1). These patterns are summarized in Table 2 and identified the serovars Huallaga of *L. noguchii* (profile I) and Trinidad of *L. santarosai* (profile J).

The combination of both enzymes, TaqI and *Tru*1I, generated 23 distinct patterns with some interesting results as follows: profile A of *Taq*I and *Tru*1I (profile AA) was species-specific and was only observed for *L. biflexa.* Profile AC was displayed by all serovars of *L. kirschneri* and by serovar Hualien of *L. terpstrae*; therefore, it is nearly species-specific. Finally, the profiles AG, FG, FE, CE, FD, BE and GE were unique to the serovars Mini, Kaup, Lanka, Szwajizak, Waskurin, Myocastoris and Maru, respectively.

Digestion with the *Sau*3AI enzyme generated nine distinct restriction patterns, which are summarized in Table 2 with the following frequencies: A, 2.94% (2); B, 22.1% (15); C, 30.9% (21); D, 4.41% (3); E, 7.35% (5); F, 16.2% (11); G, 8.82% (6); H, 5.88% (4); and I, 1.47% (1). *Sau*3AI digestion identified only the serovar Ranarum to have a serovar-specific profile. However, the combination of all three enzymes generated 30 distinct profiles, including EEE, DFD, ACC, HDD, GBB and AHH, which were specific for the serovars Whitcomb, Icterohaemorrhagiae, Hardjo, Ramisi, Semaranga, Vargonicas and Sarmin, respectively.

Finally, digestion with the enzyme *Msl*I produced only five distinct restriction patterns, which are summarized in Table 2 with the following frequencies: A, 10.3% (7); B, 20.6% (14); C, 57.4% (39); D, 10.3% (7); and E, 1.47% (1). Only the serovar Saopaulo was identified by this enzyme to have a serovar-specific profile.

The combination of the four enzymes *Taq*I, *Tru*1I, *Sau*3AI and *Msl*I generated 35 distinct profiles and identified the serovars Parameles (EFFD) and Celledoni (HDFA). In addition, this combination helped to distinguish the serovars Valbuzzi and Tropica, which had the profiles DFFC and GBCC, respectively (Table 3).

**Table 3:** Grouping of the serovars, serogroups and species of the *Leptospira* genus based on the restriction patterns generated with the four endonucleases.

Number	TaqI	Tru1I	Sau3AI	MslI	Pattern	Species/Serogroup/Serovar
1	A	A	A	A	1	*L. biflexa*/Andamana/Andamana
2	A	A	A	A	1	*L. biflexa*/Semaranga/Patoc
3	B	B	B	B	2	*L. borgpetersenii*/Autumnalis/Srebarna
5	B	B	B	B	2	*L. borgpetersenii*/Bataviae/Moldaviae
12	B	B	B	B	2	*L. borgpetersenii*/Pyrogenes/Kwale
14	B	B	B	B	2	*L. borgpetersenii*/Tarassovi/Tarassovi
4	B	C	C	B	3	*L. borgpetersenii*/Ballum/Ballum
10	B	C	C	B	3	*L. borgpetersenii*/Javanica/Javanica
6	C	D	D	B	4	*L. borgpetersenii*/Celledoni/Withcombi
7	A	E	C	C	5	*L. borgpetersenii*/Hebdomadis/Nona
16	A	E	C	C	5	*L. inadai*/Panama/Mangus
8	C	D	E	C	6	*L. borgpetersenii*/Hebdomadis/Worsfoldi
64	C	D	E	C	6	*L. weilli*/Javanica/Coxi
66	C	D	E	C	6	*L. weilli*/Tarassovi/Vughia
9	D	F	F	C	7	*L. borgpetersenii*/Icterohaemorrhagiae/Tonkini
20	D	F	F	C	7	*L. interrogans*/Autumnalis/Autumnalis
26	D	F	F	C	7	*L. interrogans*/Hebdomadis/Hebdomadis
30	D	F	F	C	7	*L. interrogans*/Pomona/Pomona
23	D	F	F	C	7	*L. interrogans*/Canicola/Canicola
25	D	F	F	C	7	*L. interrogans*/Gryppotyphosa/Valbuzzi
11	A	G	C	B	8	*L. borgpetersenii*/Mini/Mini
13	E	E	G	C	9	*L. borgpetersenii*/Sejroe/Sejroe
15	E	E	G	C	9	*L. inadai*/Canicola/Malaya
24	E	E	G	C	9	*L. interrogans*/Djasiman/Sentot
21	E	E	G	C	9	*L. interrogans*/Djasiman/Djasiman
17	F	G	H	C	10	*L. inadai*/Tarassovi/Kaup
18	E	F	F	C	11	*L. interrogans*/Australis/Australis
22	E	F	F	C	11	*L. interrogans*/Bataviae/Bataviae
19	G	B	C	C	12	*L. interrogans*/Australis/Muenchen
51	G	B	C	C	12	*L. santarosai*/Bataviae/Kobbe
53	G	B	C	C	12	*L. santarosai*/Gryppotyphosa/Canalzonae
56	G	B	C	C	12	*L. santarosai*/Mini/Georgia
61	G	B	C	C	12	*L. santarosai*/Shermani/Luis
62	G	B	C	C	12	*L. santarosai*/Tarassovi/Bakeri
59	G	B	C	C	12	*L. santarosai*/Sarmin/Weaveri
27	E	E	E	D	13	*L. interrogans*/Icterohaemorrhagiae/Icterohaemorrhagiae
28	F	E	E	C	14	*L. interrogans*/Louisiania/Lanka
29	C	E	H	C	15	*L. interrogans*/Mini/Szwajizak
31	D	B	G	C	16	*L. interrogans*/Pyrogenes/Pyrogenes
58	D	B	G	C	16	*L. santarosai*/Pyrogenes/Alexi
32	F	D	H	C	17	*L. interrogans*/Sarmin/Waskurin
33	D	F	D	C	18	*L. interrogans*/Sejroe/Hardjo
34	A	C	C	A	19	*L. kirschneri*/Australis/Ramisi
35	A	C	B	B	20	*L. kirschneri*/Bataviae/Djatzi
36	A	C	B	B	20	*L. kirschneri*/Canicola/Bafani
37	A	C	B	B	20	*L. kirschneri*/Cynopteri/Cynopteri
38	A	C	B	B	20	*L. kirschneri*/Gryppotyphosa/Gryppotyphosa
41	A	C	B	B	20	*L. kirschneri*/Pomona/Mozdok
67	A	C	B	B	20	*L. terpstrae/*Icterohaemorrhagiae/Hualien
39	A	C	B	C	21	*L. kirschneri*/Hebdomadis/Kambale
40	A	C	B	C	21	*L. kirschneri*/Icterohaemorrhagiae/Mwogolo
42	E	F	F	D	22	*L. meyeri*/Mini/Parameles
43	A	H	I	C	23	*L. meyeri*/Ranarum/Ranarum
44	H	D	D	A	24	*L. meyeri*/Semaranga/Semaranga
45	A	E	C	D	25	*L. noguchii*/Autumnalis/Fortbragg
47	A	E	C	D	25	*L. noguchii*/Panama/Panama
49	A	E	C	D	25	*L. noguchii*/Shermani/Carimagua
52	A	E	C	D	25	*L. santarosai*/Cynopteri/Tingomariensis
46	I	I	B	A	26	*L. noguchii*/Djasiman/Huallaga
48	B	E	B	D	27	*L. noguchii*/Pyrogenes/Myocastoris
50	J	B	C	C	28	*L. santarosai*/Autumnalis/Alice
54	G	E	C	A	29	*L. santarosai*/Hebdomadis/Maru
55	G	B	B	C	30	*L. santarosai*/Javanica/Vargonicas
57	G	B	C	C	31	*L. santarosai*/Pomona/Tropica
60	G	J	C	C	32	*L. santarosai*/Sejroe/Trinidad
63	H	D	F	A	33	*L. weilli*/Celledoni/Celledoni
65	A	H	H	C	34	*L. weilli*/Sarmin/Sarmin
68	H	D	F	E	35	*L. yanagawae*/Semaranga/Saopaulo

Out of sixty-eight serovars analyzed for RFLP polymorphisms in the region of the coding sequence containing the β-subunit gene of RNA polymerase, 22 serovars from nine species (32%) were identified by digestion with the enzymes *Taq*I, *Tru*1I, *Sau*3AI and *Msl*I (Table 3), and the other 46 strains were clustered into 13 groups with two to seven serovars.

**Figure 2:**
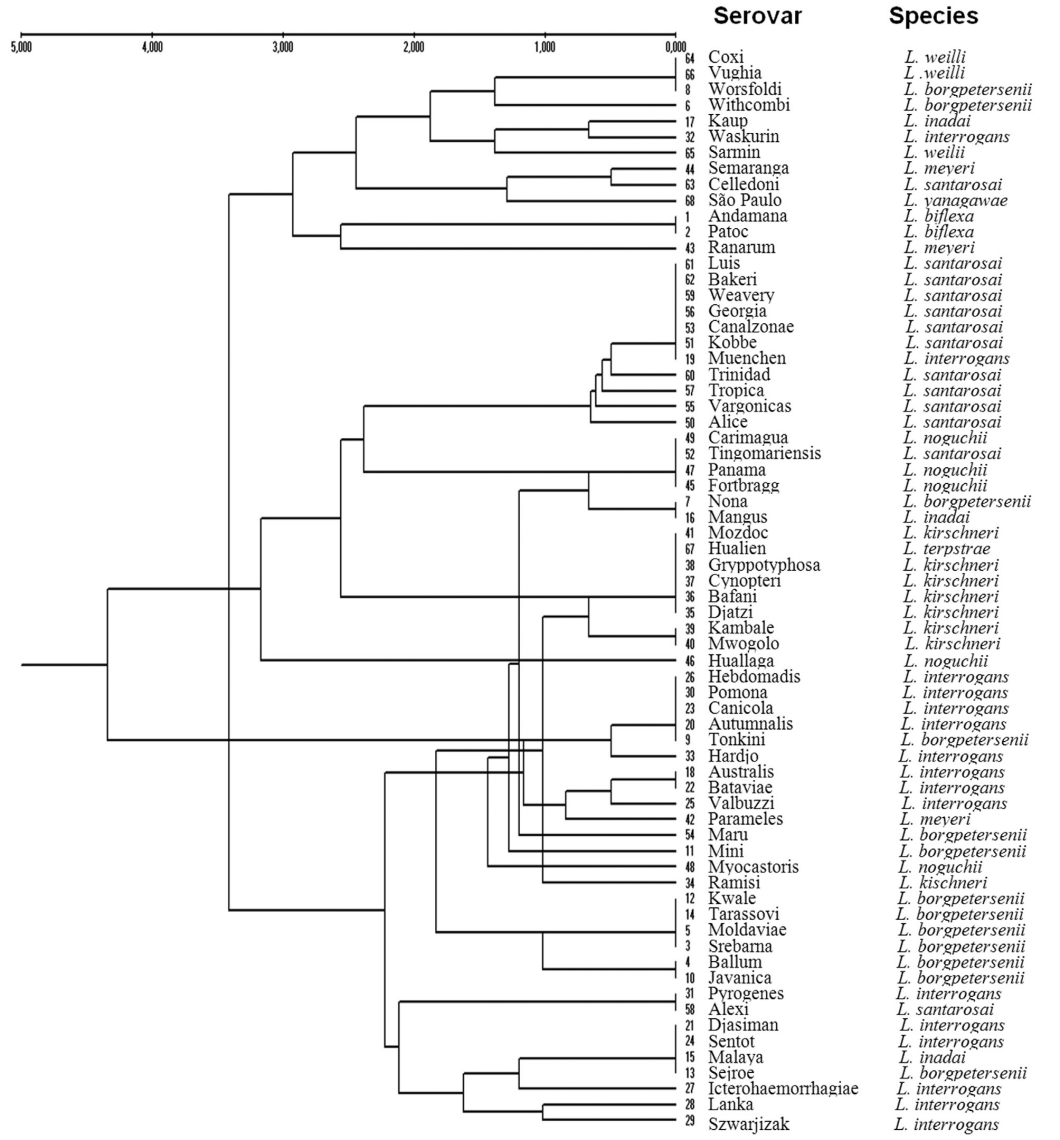
Dendrogram constructed by joint analysis of the bands generated by the restriction endonucleases *Taq*I, *Tru*1I, *Sau*3AI, and *Msl*I.

A dendrogram obtained from a matrix constructed with the results from the fragments generated by PCR-RFLP with the four restriction endonucleases (Figure 2) showed clustering of the sixty-eight reference serovars. Several of the tested strains appeared to be distant from others of the same species in relation to the current taxonomic classification. The serovar Kaup (*L. inadai*) was grouped with the serovar Waskurin (*L. interrogans*); the serovar Ranarum (*L. meyeri*) was similar to the nonpathogenic *L. biflexa*; the serovar Muenchen (*L. interrogans*) clustered with those of *L. santarosai*; the serovar Nona (*L. borgpetersenii*) was closer to the serovar Mangus (*L. inadai*); the serovar Hualien (*L. terpstrae*) grouped with the those of *L. kirschneri*; the Huallaga and Myocastoris serovars (*L. noguchii*) were located in different branches; the serovar Tonkini (*L. borgpetersenii*) grouped with the majority of those of *L. interrogans*; the serovar Ramisi (*L. kirschneri*) was closer to those of *L. borgpetersenii*; and the serovar Alexi (*L. santarosai*) was grouped with those of Djasiman, Pyrogenes and Sentot (*L. interrogans*), Malaya (*L. inadai*) and Sejroe (*L. borgpetersenii*).

## Discussion

The correlation between the serological and genotypic classifications of leptospires is low, and identification is complicated because the same serovar can be distributed among different species (Ahmed *et al.*, 2012; Balamurugan *et al.*, 2013). It is assumed that this lack of correlation between species and serovars is the result of horizontal transference between species of the genes that determine serotypes, but the basis of this transference, which is responsible for exchanging genetic determinants, is still unknown (Cerqueira and Picardeau, 2009). A single base difference differentiated many strains of *L. interrogans* and *L. kirschneri*; therefore, phylogenetic representation may be less meaningful than sequence identities at variable positions (Cerqueira and Picardeau, 2009).

The aim of this work was to identify *Leptospira* strains at the serovar level by performing PCR-RFLP to amplify a 600-bp fragment of the coding sequence of the β subunit of the RNA polymerase gene. The *rpo*B gene has been widely studied in other organisms and is considered by many researchers to be more useful than the 16S ribosomal RNA gene for the differentiation of bacterial species (La Scola *et al.*, 2006a; Ahmed *et al.*, 2006; Macheras *et al.*, 2011; Ahmed *et al.*, 2012). In addition, twenty-five sequences of the rpoB gene of *Leptospira* are already available in databases, thereby facilitating access and minimizing project costs.

In a previous report, La Scola *et al.* (2006a) have compared similarities in the *rrs* and *rpo*B genes between different *Leptospira* serovars. Using the *rpo*B gene, they were able to effectively distinguish 11 of 19 serovars tested, differentiating them from other species and showing greater numbers of polymorphisms in both genes, leading to the conclusion that the rpoB gene could distinguish species with a higher number of differences between base pairs.

In this study, 68 *Leptospira* serovars were analyzed for polymorphisms in a specific region of the *rpo*B gene using the endonucleases *Taq*I, *Tru*1I, *Sau*3AI and *Msl*I. These enzymes were selected after *in silico* restriction digestion of the *rpo*B sequences deposited in GenBank. We were able to identify 22 strains from nine species at the serovar level (32%). The rpoB gene has been widely used as an alternative tool in the phylogeny and identification of different species of bacteria, such as *Coxiella burnetii* (Mollet *et al.*, 1998), *Afipia* (Khamis *et al.*, 2003), *Mycoplasma* (Kim *et al.*, 2003), *Corynebacterium* (Khamis *et al.*, 2004), *Acinetobacter* (La Scola *et al.*, 2006b), *Mycobacterium* (Adekambi *et al.*, 2006; Ben *et al.*, 2008), *Halobacterium* (Minegishi *et al.*, 2010) and *Cyanobacteria* (Gaget *et al.*, 2011).

In a recent study, the rpoB gene has been successfully used to identify or detect *Leptospira* species in animals and humans in India (Balamurugan *et al.*, 2013). Because each *Leptospira* serovar is associated with specific host symptoms, their identification is essential for the development of epidemiological studies (Cerqueira and Picardeau, 2009, Li *et al.*, 2009).

Clustering analysis of the results of this study correctly grouped 22 serovars by species. Considerable similarities in the analyzed genomic region were observed among all serovars. Analysis of dendrograms constructed from the results of each restriction enzyme and from the collective results for all of the enzymes showed the formation of clusters, for which serovars of various species had identical profiles. The groups formed by the *rpo*B gene profiles showed varying degrees of similarity and clade formation. Based on this, similar banding patterns were observed among the serovars Mangus, Nona, Alexi, Pyrogenes, Sentot, Malaya and Sejroe, despite the fact that they belonged to different species. These findings are in accordance with similar dendrogram analyses reported previously (Perolat *et al.*, 1998; Morey *et al.*, 2006; Cerqueira and Picardeau, 2009; Balamurugan *et al.*, 2013), showing similar cluster formations and variations in serovar-species grouping.

The addition of new enzymes for the production of additional profiles should clarify the positions of other serovars. Still, these results suggest that the use of this technique to assess gene sequences may reveal a precise *sensu stricto* classification of these serovars.

Molecular techniques have been used for the characterization of *Leptospira* isolates; however, most can only make identifications to the species level (Galloway and Levett, 2010), such as 16S rRNA sequence analysis (Morey *et al.*, 2006), RFLP (Li *et al.*, 2009) and MLST (Boonsilp *et al.*, 2013). PFGE has demonstrated the reliable and reproducible identification of *Leptospira* at the serovar level (Galloway and Levett, 2010). These approaches have greatly contributed to a revolution in both *Leptospira* detection and characterization (Ahmed *et al.*, 2012). On the other hand, the molecular tools described so far for the characterization of *Leptospira* suffer from significant drawbacks. For example, PFGE, RFLP, and REA require large quantities of purified DNA, have low levels of discrimination, produce data that is difficult to interpret, suffer from a lack of reproducibility and require specialized equipment (Ahmed *et al.*, 2006).

Notably, the 16S rRNA gene has been considered the gold standard in molecular surveys of bacterial and archaeal diversity, but it has several disadvantages as follows: it is often present in multiple copies, has little resolution below the species level and cannot be readily interpreted in an evolutionary framework (Vos *et al.*, 2012).

The main advantages of the use of the *rpoB* gene over the 16S rRNA gene are as follows: (i) it is universally present in all prokaryotes; (ii) it typically occurs in a single-copy, essential protein-encoding gene, and sequence errors can be readily identified and removed if they introduce disruptions in the reading frame; (iii) it possesses both slowly and quickly evolving regions, enabling the design of probes and primers of differing specificities; (iv) it has a housekeeping function, making it less susceptible to some forms of lateral gene transfer; and (v) it is large enough in size to contain phylogenetic information, even after the removal of regions that are difficult to align (Case *et al.*, 2007; Vos *et al.*, 2012).

Our findings "in vitro" indicate that the PCR-RFLP technique is a powerful and reproducible test that may be used as a complement or alternative tool to assess the distribution of *Leptospira* strains within species. Additionally, we recommend the use of PCR-RFLP with *in silico* digestion of the polymorphic sequences of other conserved genes already deposited in GenBank as a promising technique for the genomic classification of the *Leptospira* genus.

## Conclusion

This study demonstrated that PCR-RFLP is practical and efficient, enabling the differentiation of species and serovars with good discriminatory power, reproducibility and easily interpretable results. In addition, this method is cost-effective for most research laboratories. This technique has also been shown to be suitable for phylogenetic studies and the classifications of species, serovars and strains. The selected 600-bp polymorphic sequence of the *rpo*B gene produced restriction profiles that allowed for the accurate and timely identification of 32% of the 68 tested strains. We demonstrated that this approach achieves the stated purpose and that serological typing is unreliable for the classification of pathogenic *Leptospira*. However, additional studies should be undertaken to reclassify these serovars within the species with which they have greater genotypic affinities based on analysis of hypervariable regions of multiple housekeeping genes and especially to investigate whether the clinical leptospirosis symptoms induced by these serovars are presented according to the species with which they are most phylogenetically related.
